# Anti-inflammatory effects of flavonoids and phenylethanoid glycosides from *Hosta plantaginea* flowers in LPS-stimulated RAW 264.7 macrophages through inhibition of the NF-κB signaling pathway

**DOI:** 10.1186/s12906-022-03540-1

**Published:** 2022-03-03

**Authors:** Li Yang, Junwei He

**Affiliations:** 1 College of Pharmacy, Jiangxi University of Chinese Medicine, Nanchang, 330004 China; 2 Research Center of Natural Resources of Chinese Medicinal Materials and Ethnic Medicine, Jiangxi University of Chinese Medicine, No. 1688, Meiling Road, Nanchang, 330004 China

**Keywords:** *Hosta plantaginea* flowers, Anti-inflammatory, NF-κB, Flavonoid, Phenylethanoid glycoside

## Abstract

**Background:**

The flower of *Hosta plantaginea* (Lam.) Aschers has traditionally been used in China as an important Mongolian medicine for the treatment of inflammatory diseases with limited scientific evidence. In previous studies, 16 flavonoids and 3 phenylethanoid glycosides (**1**–**19**) were isolated from the ethanolic extract of *H. plantaginea* flowers. Nevertheless, the anti-inflammatory effects of these constituents remain unclear. In the present study, the anti-inflammatory effects of these 19 constituents and their underlying mechanisms were assessed in lipopolysaccharide (LPS)-stimulated RAW 264.7 macrophages.

**Methods:**

The viability of RAW 264.7 macrophages was detected by Cell Counting Kit-8 (CCK-8) assay. Meanwhile, nitric oxide (NO) production was measured by Griess assay, while the secretion of tumor necrosis factor α (TNF-α), prostaglandin E2 (PGE2), interleukin 1β (IL-1β) and IL-6 in LPS-induced macrophages was determined by enzyme-linked immunosorbent assay (ELISA). Furthermore, the protein expression of nuclear factor kappa B (NF-κB) p65 and phosphorylated NF-κB p65 was evaluated by Western blot analysis.

**Results:**

All constituents effectively suppressed excessive NO production at a concentration of 40 μM with no toxicity to LPS-induced RAW 264.7 macrophages. Among them, five flavonoids (**1**, **4**–**6** and **15**) and one phenylethanoid glycoside (**17**) remarkably prevented the overproduction of NO with median inhibitory concentration (IC_50_) values in the range of 12.20–19.91 μM. Moreover, compounds **1**, **4**–**6**, **15** and **17** potently inhibited the secretion of TNF-α, PGE2, IL-1β and IL-6, and had a prominent inhibitory effect on the down-regulation of the phosphorylated protein level of NF-κB p65.

**Conclusion:**

Taken together, compounds **1**, **4**–**6**, **15** and **17** may be useful in managing inflammatory diseases by blocking the NF-κB signaling pathway and suppressing the overproduction of inflammatory mediators.

**Supplementary Information:**

The online version contains supplementary material available at 10.1186/s12906-022-03540-1.

## Background

Inflammation is an innate, automatic and complex immune system response of the body to tissue injury, infection or irritation caused by bacteria, toxins and other substances [1–4]. However, excessive inflammation could contribute to the pathogenesis of various acute and chronic inflammation-related diseases [1–4]. Therefore, controlling inflammatory overexpression is a vital tool for the prevention and treatment of inflammatory diseases.

Macrophages are one of the most important inflammatory and immune cells, playing a crucial role in the inflammatory process [1–4]. In particular, lipopolysaccharide (LPS) is an endotoxin that strongly triggers macrophages to activate the NF-κB signaling pathway and produce numerous inflammatory mediators, such as NO, TNF-α, PGE2, IL-1β and IL-6 [3–7]. Therefore, the LPS-stimulated RAW 264.7 macrophage is commonly used as a classical inflammatory cell model to evaluate the anti-inflammatory activity and underlying mechanisms of action of drugs [3–7]. Furthermore, NF-κB is regarded as an important transcription factor in the pathogenesis of inflammatory diseases, and its activation positively regulates the expression of inflammatory mediators [3–7]. Hence, inhibition of NF-κB signaling pathway can be considered as an important target for the prevention and treatment of inflammatory diseases.

Natural products from medicinal plants, especially those derived from traditional folk medicine, are vital sources of anti-inflammatory therapy [1–7]. As one of them, *Hosta plantaginea* (Lam.) Aschers is an important traditional medicinal plant, mainly distributed in temperate and sub-tropical zones of Asia [8]. In china, the flower of *H. plantaginea*, also known as “Yu-zan-hua”, has been widely used for thousands of years as a very important traditional Mongolian medicine for the treatment of inflammatory diseases, such as sore throat, acute and chronic laryngopharyngitis [8–10]. Its crude extract exhibits anti-inflammatory, anti-tumor, anti-viral, antimicrobial and other effects [8–10]. In our previous study, 16 flavonoids (**1**–**16**) and 3 phenylethanoid glycosides (**17**–**19**) comprising kaempferol (**1**), astragalin (**2**), kaempferol-7-*O*-*β*-D-glucopyranoside (**3**), kaempferol-3,7-di-*O*-*β*-D-glucopyranoside (**4**), kaempferol-3-*O*-sophoroside (**5**), plantanone A (**6**), kaempferol-3-*O*-*β*-D-[*β*-D-glucopyranosyl-(1 → 2)-glucopyranoside]-7-*O*-*β*-D-glucopyranoside (**7**), kaempferol-3-*O*-rutinoside-7-*O*-glucopyranoside (**8**), kaempferol-3-*O*-*α*-L-rhamnopyranosyl-(1 → 6)-*β*-D-glucopyranosyl-(1 → 2)-*β*-D-glucopyranoside (**9**), kaempferol-3-*O*-*β*-D-glucopyranosyl-(1 → 2)-[*α*-L-rhamnopyranosyl-(1 → 6)]-*β*-D-glucopyranoside (**10**), kaempferol-3-*O*-rutinoside (**11**), plantanone B (**12**), plantanone D (**13**), naringenin (**14**), dihydrokaempferol (**15**), hostaflavanone A (**16**), phenethyl-*O*-*β*-D-glucopyranoside (**17**), phenethanol-*β*-gentiobioside (**18**) and phenethyl-*O*-rutinoside (**19**), were isolated from the ethanolic extract of *H. plantaginea* flowers [11–15]. Of these, all constituents except **13**–**15** exhibited potential inhibitory effect on cyclooxygenase 2 (COX-2) in vitro. Nevertheless, the anti-inflammatory function of these 19 constituents and their underlying mechanisms in cells have not been deeply studied.

To reveal the underlying mechanisms of *H. plantaginea* as a treatment for inflammatory diseases, the anti-inflammatory effects of these 19 constituents isolated from this traditional Chinese medicine were evaluated in LPS-stimulated RAW 264.7 macrophages. We also hope to screen the most effective anti-inflammatory candidates from the flowers of *H. plantaginea*.

## Materials and methods

### Chemicals and reagents

Our previous studies reported the isolation and identification of 16 flavonoids and 3 phenylethanoid glycosidescomprising kaempferol (**1**), astragalin (**2**), kaempferol-7-*O*-*β*-D-glucopyranoside (**3**), kaempferol-3,7-di-*O*-*β*-D-glucopyranoside (**4**), kaempferol-3-*O*-sophoroside (**5**), plantanone A (**6**), kaempferol-3-*O*-*β*-D-[*β*-D-glucopyranosyl-(1 → 2)-glucopyranoside]-7-*O*-*β*-D-glucopyranoside (**7**), kaempferol-3-*O*-rutinoside-7-*O*-glucopyranoside (**8**), kaempferol-3-*O*-*α*-L-rhamnopyranosyl-(1 → 6)-*β*-D-glucopyranosyl-(1 → 2)-*β*-D-glucopyranoside (**9**), kaempferol-3-*O*-*β*-D-glucopyranosyl-(1 → 2)-[*α*-L-rhamnopyranosyl-(1 → 6)]-*β*-D-glucopyranoside (**10**), kaempferol-3-*O*-rutinoside (**11**), plantanone B (**12**), plantanone D (**13**), naringenin (**14**), dihydrokaempferol (**15**), hostaflavanone A (**16**), phenethyl-*O*-*β*-D-glucopyranoside (**17**), phenethanol-*β*-gentiobioside (**18**) and phenethyl-*O*-rutinoside (**19**) from the ethanolic extract of *H. plantaginea* flowers, a plant (Voucher specimen number: YZH201409) which was identified by professor Guoyue Zhong (Jiangxi University of Chinese Medicine, Nanchang, China) [11–15]. Moreover, the purity of each compound was greater than 97% as determined by high performance liquid chromatography analysis.

Fetal bovine serum (FBS) was obtained from Hyclone (Logan, UT, USA). Lipopolysaccharide (LPS, *Escherichia coli* serotype 0111: B4, L5293) was purchased from Sigma-Aldrich (St. Louis, MO, USA). Dulbecco’s Modified Eagle Medium (DMEM) and trypsase were procured from GIBCO (Grand Island, NY, USA). Penicillin-streptomycin was purchased from Sigma-Aldrich (St. Louis, MO, USA). CCK-8 and radioimmunoprecipitation assay (RIPA) lysis buffer were acquired from Beyotime Institute of Biotechnology (Shanghai, China). Murine ELISA kits for TNF-α, IL-1β and IL-6 were acquired from R&D Systems (Minnesota, USA). The murine ELISA kit for PGE2 was obtained from Westang (Shanghai, China). Antibodies against phos-NF-κB p65 (Ser536) and NF-κB p65 were purchased from Cell Signaling Technology (Boston, USA).

### Cell culture

Murine RAW 264.7 macrophages were purchased from the American Tissue Culture Collection (Manassas, USA). These cells were incubated in DMEM supplemented with 10% FBS, 100 U/mL penicillin and 100 U/mL streptomycin in humidified 5% carbon dioxide (CO_2_) at 37 °C [16, 17].

### Effects of 19 constituents on the viability of RAW 264.7 macrophages

The effects of 19 constituents on the viability of RAW 264.7 macrophages were assessed by CCK-8 assay [18, 19]. Prior to treatment, RAW 264.7 macrophages (5 × 10^3^ cells/well) were seeded into 96-well plates and incubated for 24 h. All cultured cells were treated with or without 19 compounds at a concentration of 40 μM at 37 °C for 24 h. After incubation, 10 μL of CCK-8 solution was added to each well and incubated at 37 °C. After 2 h, the absorbance of each well was measured at 450 nm in a microplate reader. Cell viability was calculated using the following formular:

Cell viability (%) = A_sample_/A_control_ × 100, where A_sample_ and A_control_ are the absorbance of cells treated with the compound and untreated cells, respectively. Moreover, the latter is expressed as 100% cell viability.

### Effects of 19 constituents on LPS-induced NO production by the Griess method

The effects of 19 constituents on NO production in LPS-induced RAW 264.7 macrophages were determined using the Griess method [18, 19]. Prior to treatment, RAW 264.7 macrophages (5 × 10^3^ cells/well) were seeded into 96-well plates and incubated for 24 h. All cultured cells were treated with or without 19 compounds at a concentration of 40 μM at 37 °C. After 1 h, cells were incubated with or without LPS at a concentration of 1 μg/mL at 37 °C for 24 h. After incubation, 50 μL of each supernatant solution was collected and mixed with equal volumes of Griess Reagent I and Griess Reagent II, respectively. The absorbance (A) was measured at 540 nm after 10 min of incubation at room temperature. The standard concentration of sodium nitrite was used to calculate the nitrite concentration. Moreover, the NO inhibition rate (%) = (A_LPS_ − A_LPS + sample_)/(A_LPS_ − A_control_) × 100, where A_LPS_, A_LPS + sample_, and A_control_ are the absorbance of LPS model group, LPS + sample group, and DMEM group, respectively.

Subsequently, all compounds with NO inhibition rates greater than 50%, including **1**, **4**–**7**, **11**, **13**–**15** and **17**, were further investigated in accordance with the method described above to determine the NO levels at concentrations of 1.25, 2.5, 5, 10 and 20 μM, respectively.

### Effects of compounds 1, 4–6, 15 and 17 on LPS-induced pro-inflammatory cytokine production by ELISA assay

The logarithmic growth phase of RAW 264.7 macrophages was inoculated in 96-well plates at a density of 5 × 10^3^ cells/well. After 24 h of incubation, these cells were pretreated with respective concentrations of 20 μM of each compound (**1**, **4**–**6**, **15** and **17**) for 1 h, followed by the addition of LPS (1 μg/mL) for 24 h. Finally, 50 μL of each supernatant solution was taken to measure the concentrations of TNF-α, PGE2, IL-1β and IL-6, using the corresponding commercially available murine ELISA kits in accordance with the manufacturer’s instructions [16, 17].

### Effects of compounds 1, 4–6, 15, and 17 on LPS-induced NF-kB activation by Western blotting

RAW 264.7 macrophages were seeded and pretreated with respective concentrations of 20 μM of each compound (**1**, **4**–**6**, **15** and **17**) for 1 h, followed by the addition of LPS (1 μg/mL) for 24 h. Subsequently, the total protein was extracted by resuspending the cells in RIPA lysis buffer. In addition, protein concentrations were measured by a Bicinchoninic acid (BCA) assay kit. Proteins were separated using SDS-PAGE gels and then electroplated onto a PVDF membrane, which was blocked with 5% skim milk for 1 h at room temperature in Tris-buffered saline-Tween (TBST). Membranes were washed three times with TBST and then incubated overnight at 4 °C in diluted (1:1000) primary antibody solution (anti-NF-κB p65 or anti-phosphorylated-NF-κB p65). After washing three times with TBST, the membranes were incubated with a 1:5000 dilution of HRP-conjugated secondary antibody for 1 h at room temperature. The immunoreactive bands were determined by densitometry and quantified using a Bio-Rad auto-developer (Bio-Rad, California, USA). All results are expressed as relative ratios to the reference protein GAPDH [16, 17].

### Statistical analysis

All results were reproduced in triplicate and expressed as mean ± standard deviation (SD). Multiple data sets were compared using one-way analysis of variance (ANOVA) followed by Tukey’s test using GraphPad Prism 6, and *P* < 0.05 was considered significant.

## Results

### Absence of negative effect of all constituents on RAW 264.7 macrophage viability

The cell viability of these 19 constituents in RAW 264.7 macrophages was performed using the CCK-8 method. As shown in Table 1, all constituents at a concentration of 40 μM showed no toxicity to RAW 264.7 macrophages after 24 h of treatment (*p* > 0.05). Accordingly, subsequent experiments were conducted with 19 constituents at concentrations not exceeding 40 μM.

**Table 1:** Effects of 16 flavonoids (**1**–**16**) and 3 phenylethanoid glycosides (**17**–**19**) on cell viability of RAW 264.7 macrophages^a^

Compound	Cell viability (%)	Compound	Cell viability (%)
Control	100 ± 10.32	**10**	93.76 ± 5.06
**1**	92.36 ± 3.96	**11**	90.87 ± 5.45
**2**	95.01 ± 1.86	**12**	91.93 ± 8.04
**3**	91.12 ± 5.28	**13**	91.84 ± 1.11
**4**	93.38 ± 6.34	**14**	92.97 ± 6.19
**5**	93.48 ± 5.33	**15**	91.31 ± 5.38
**6**	96.89 ± 1.04	**16**	92.56 ± 1.97
**7**	94.03 ± 4.52	**17**	92.21 ± 3.48
**8**	95.48 ± 3.54	**18**	92.51 ± 5.62
**9**	90.70 ± 3.71	**19**	92.30 ± 9.38

^a^Values are mean ± SD of three independent experiments (*n* = 3). One-way ANOVA followed by Tukey’s test with GraphPad Prism 6

### Reduction of NO production in LPS-α-induced RAW 264.7 macrophages by all constituents

**Fig. 1:**
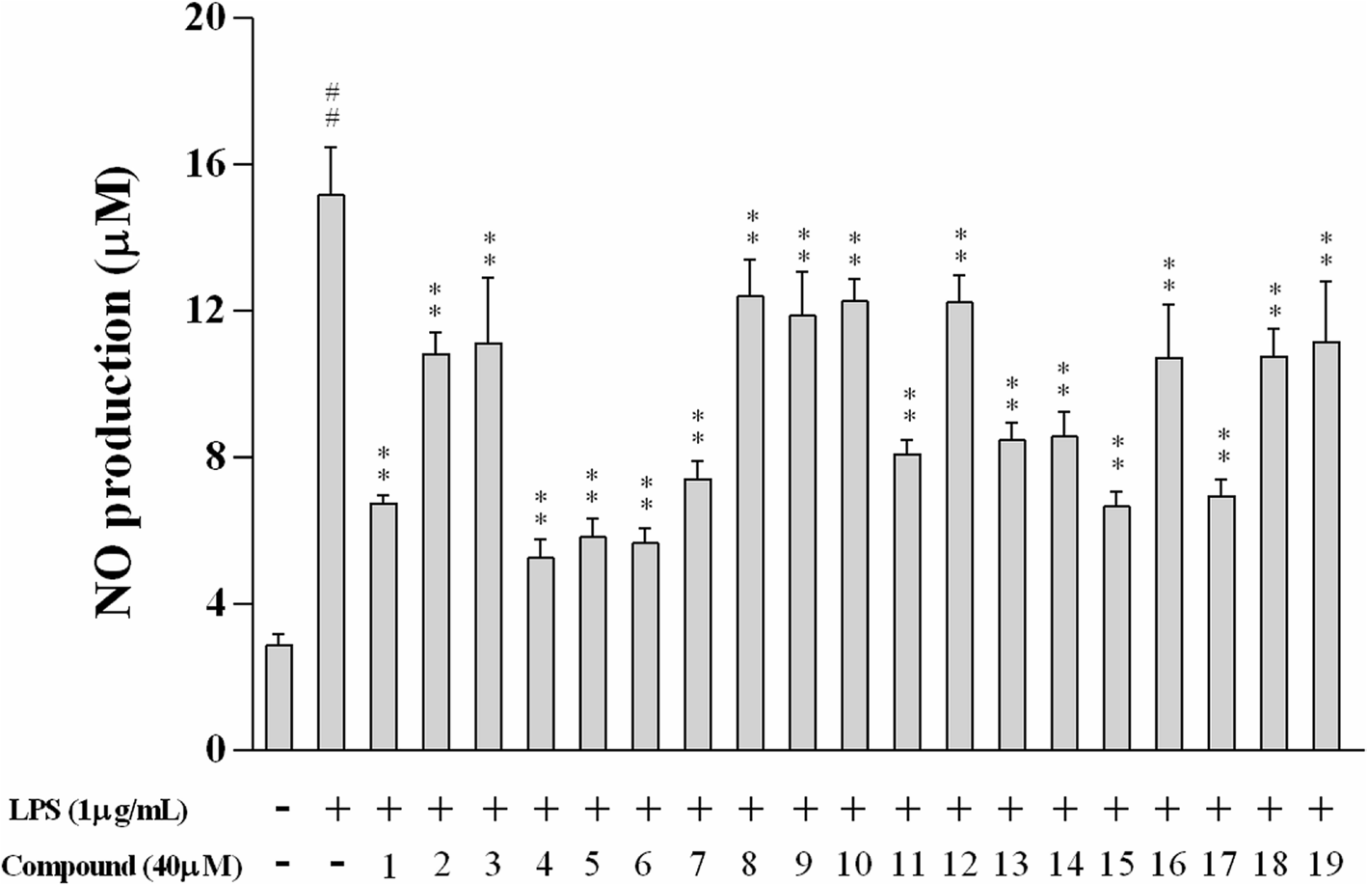
Effects of 16 flavonoids (**1**–**16**) and 3 phenylethanoid glycosides (**17**–**19**) on NO production in LPS-stimulated RAW 264.7 macrophages. All data from three independent experiments are expressed as mean ± SD. ^##^*p* < 0.01 vs. culture medium-only control group; ^**^p < 0.01 vs. LPS-only model group. One-way ANOVA, followed by Tukey’s test using GraphPad Prism 6

As illustrated in Fig. 1, LPS induced a dramatic production of NO, which was prominently reduced by 19 constituents at a concentration of 40 μM, and their NO inhibition rates exceeded 50% except for compounds **2**, **3**, **8**–**10**, **12**, **16**, **18** and **19**.

**Fig. 2:**
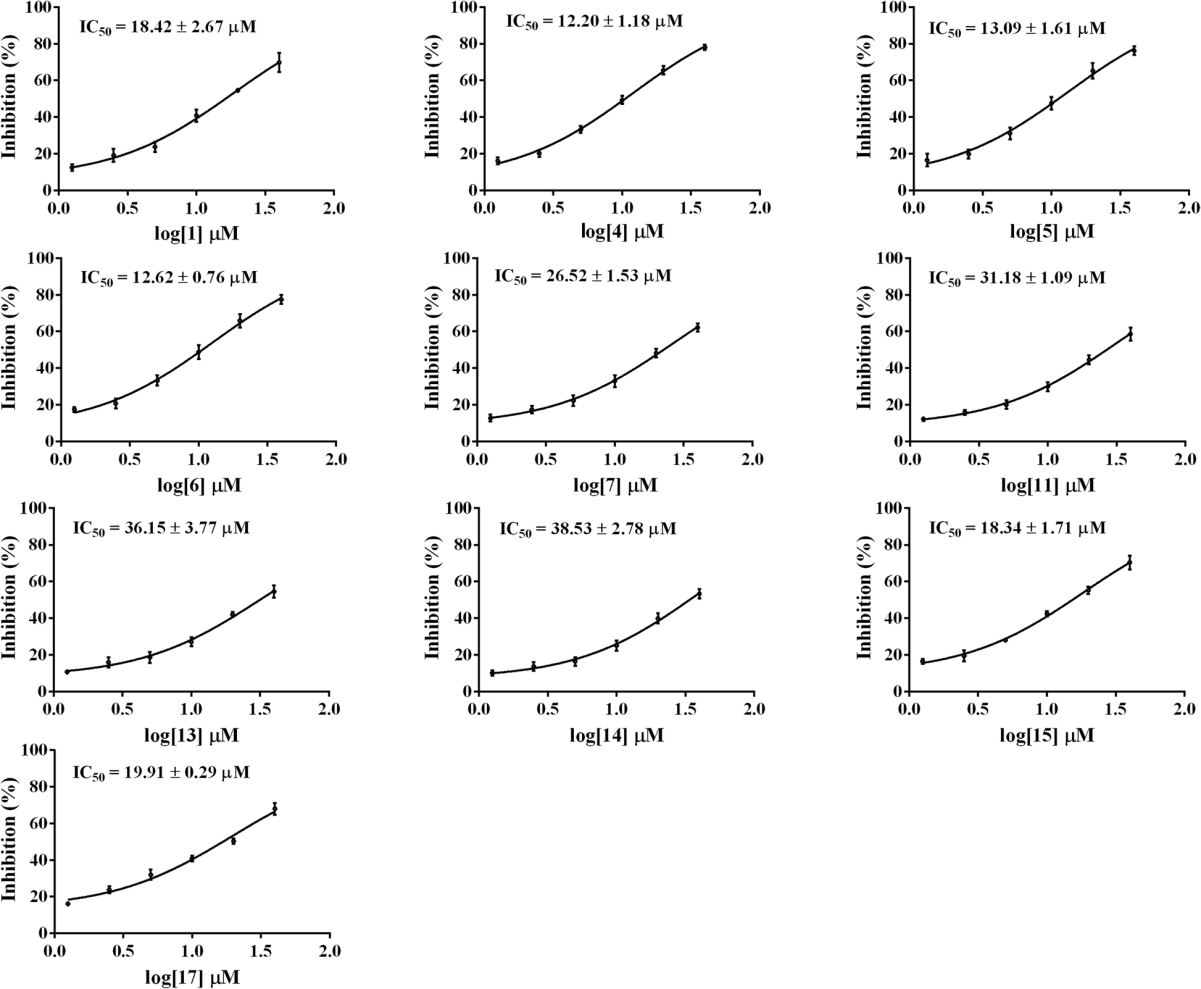
Dose-response curves for compounds **1**, **4**–**7**, **11**, **13**–**15** and **17** in the NO inhibition assay (*n* = 3)

Subsequently, 9 flavonoids (**1**, **4**–**7**, **11** and **13**–**15**) and one phenylethanoid glycoside (**17**) were further evaluated for their inhibitory effects on NO production in LPS-stimulated RAW 264.7 macrophages. As a result, these ten constituents may remarkably reduce NO production in a concentration-dependent manner with half-maximal inhibitory concentration (IC_50_) values in the range of 12.20–38.53 μM (Table 2 and Fig. 2). Among them, compounds **1**, **4**–**6**, **15** and **17** showed the strongest effect on NO production with IC_50_ values not exceeding 20 μM. As such, compounds **1**, **4**–**6**, **15** and **17** were further evaluated for their anti-inflammatory effects and underlying mechanisms in LPS-stimulated RAW 264.7 macrophages.

**Table 2:** The IC_50_ values of 16 flavonoids (**1**–**16**) and 3 phenylethanoid glycosides (**17**–**19**) on NO production in LPS-stimulated RAW 264.7 macrophages^a^

Compound	IC_50_ (μM)	Compound	IC_50_ (μM)
**1**	18.42 ± 2.67	**11**	31.18 ± 1.09
**2**	> 40	**12**	> 40
**3**	> 40	**13**	36.15 ± 3.77
**4**	12.20 ± 1.18	**14**	38.53 ± 2.78
**5**	13.09 ± 1.61	**15**	18.34 ± 1.71
**6**	12.62 ± 0.76	**16**	> 40
**7**	26.52 ± 1.53	**17**	19.91 ± 2.01
**8**	> 40	**18**	> 40
**9**	> 40	**19**	> 40
**10**	> 40		

^a^Values are mean ± SD of three independent experiments (*n* = 3)

Based on the above results, 9 flavonoids comprising **1**, **4**–**7**, **11** and **13**–**15**, as well as one phenylethanoid (**17**), were identified as the bioactive phytochemicals contributing to anti-inflammatory activity against NO production in LPS-stimulated RAW 264.7 macrophages. Importantly, compounds **1**, **4**–**6**, **15** and **17** showed the highest efficacy against NO inhibition with IC_50_ values less than 20 μM, and were further chosen to explore the anti-inflammatory mechanism.

### Inhibiting the release of TNF-α, PGE2, IL-1β and IL-6 in LPS-stimulated RAW 264.7 macrophages by compounds 1, 4–6, 15 and 17

**Fig. 3:**
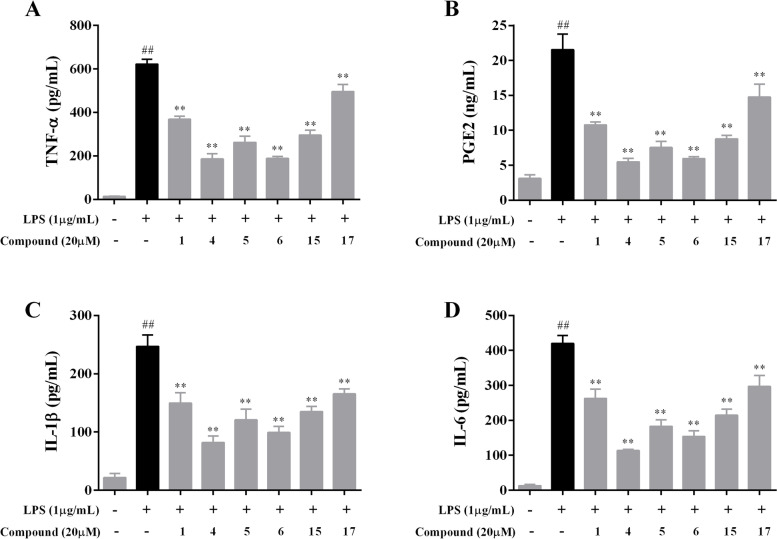
Effects of compounds **1**, **4**–**6**, **15** and **17** on the production of TNF-α (**A**), PGE2 (**B**), IL-1β (**C**) and IL-6 (**D**) in LPS-stimulated RAW 264.7 macrophages. All data from three independent experiments are expressed as mean ± SD. ^##^*p* < 0.01 vs. culture medium-only control group; ^**^p < 0.01 vs. LPS-only model group. One-way ANOVA, followed by Tukey’s test using GraphPad Prism 6

To determine whether compounds **1**, **4**–**6**, **15** and **17** affected the secretion of pro-inflammatory cytokines (including TNF-α, PGE2, IL-1β and IL-6) in LPS-stimulated RAW 264.7 macrophages, an ELISA method was performed. As depicted in Fig. 3, the levels of TNF-α, PGE2, IL-1β and IL-6 were prominently increased after LPS (1 μg/mL) treatment compared to the control group (*p* < 0.01). In contrast, treatment with compounds **1**, **4**–**6**, **15** and **17** at a concentration of 20 μM significantly reduced the levels of TNF-α, PGE2, IL-1β and IL-6 compared to the LPS group (*p* < 0.01).

### Blocking NF-kB signaling pathway in LPS-stimulated RAW 264.7 macrophages by compounds 1, 4–6, 15 and 17

**Fig. 4:**
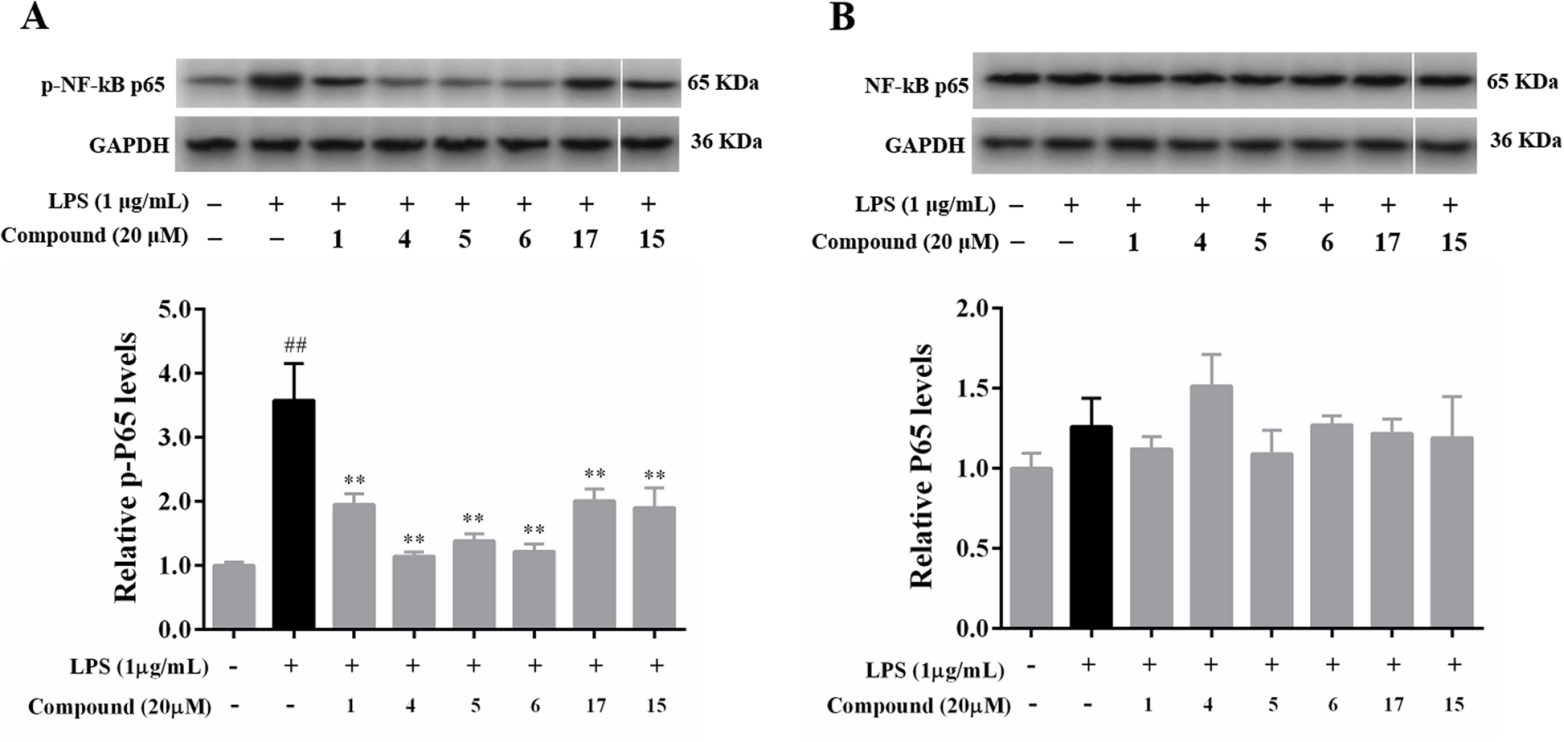
Effects of compounds **1**, **4**–**6**, **15** and **17** on NF-kB activation (**A**-phosphorylated p65 and **B**-total p65) in LPS-stimulated RAW 264.7 macrophages. All data from three independent experiments are expressed as mean ± SD. ^##^*p* < 0.01 vs. culture medium-only control group; ^**^p < 0.01 vs. LPS-only model group. One-way ANOVA, followed by Tukey’s test using GraphPad Prism 6

To determine the underlying anti-inflammatory mechanism of compounds **1**, **4**–**6**, **15** and **17**, the protein levels of NF-κB p65 and phosphorylated NF-κB p65 were examined by Western blot analysis. As shown in Fig. 4, the protein expression of phosphorylated NF-κB p65 was significantly upregulated in LPS-treated RAW 264.7 macrophages compared to untreated cells (*p* < 0.01). Phosphorylation of NF-κB p65 levels was found to be significantly lower in the treatment groups of compounds **1**, **4**–**6**, **15** and **17** at a concentration of 20 μM compared to the LPS group (*p* < 0.01) (Supplementary Material).

Based on the above evidence, compounds **1**, **4**–**6**, **15** and **17** are the most potent anti-inflammatory constituents that suppress the NF-κB signaling pathway, which results in a reduction in the secretion levels of NO, TNF-α, PGE2, IL-1β and IL-6 in LPS-stimulated RAW 264.7 macrophages.

## Discussion

In China, the flower of *H. plantaginea* is commonly used as an empirical treatment for inflammatory diseases with very limited scientific validation [8]. Crude extracts of *H. plantaginea* have been evaluated for their traditional pharmacological effects such as anti-inflammatory, anti-tumor, anti-viral, antimicrobial effects, etc. [8–10]. Although numerous phytochemicals with anti-inflammatory, anti-tumor, anti-acetylcholinesterase, and anti-viral activities have been reported [8, 9], the anti-inflammatory effects and underlying mechanisms of action of constituents derived from the flowers of *H*. *plantaginea* have not been fully explored. Furthermore, flavonoids and phenylethanoid glycosides are two major classes of phytochemicals from medicinal plants with various biological effects, such as anti-inflammatory and antioxidant [1, 11, 13, 20]. In our previous studies, 16 flavonoids (**1**–**16**) and 3 phenylethanoid glycosides (**17**–**19**), some of which have potential anti-inflammatory activity against COX-2, were isolated and identified from the ethanolic extract of *H. plantaginea* flowers [11–15]. Furthermore, COX-2 is a critical enzyme involved in the process of inflammatory responses and inflammatory diseases [11, 21–28], which suggests that the aforementioned 19 constituents may have anti-inflammatory effects. Although kaempferol (compound **1**) [29, 30], astragalin (compound **2**) [30], kaempferol-3-*O*-rutinoside (compound **11**) [31], and dihydrokaempferol (compound **15**) [32] have been shown to exhibit anti-inflammatory effects by suppressing the secretion of inflammatory cytokines in cells, their underlying molecular mechanisms remain unclear. Furthermore, naringenin (compound **14**) at concentrations of 40, 60 and 80 μM possessed anti-inflammatory effect via inhibition NF-κB and MAPKs pathways in BV2 microglia [33]. Taken together, the anti-inflammatory effects of these 19 constituents isolated from *H. plantaginea* flowers and their underlying mechanisms are still poorly understood in cellular model.

In the present study, the anti-inflammatory activities of 19 constituents isolated from *H. plantaginea* flowers were evaluated in LPS-stimulated RAW 264.7 macrophages. As a result, 5 flavonoids (**1**, **4**–**6** and **15**) and one phenylethanoid glycoside (**17**) exhibited strong anti-inflammatory effects by blocking the NF-κB signaling pathway and suppressing NO, TNF-α, PGE2, IL-1β and IL-6 production at a concentration of 20 μM.

LPS promotes the inflammatory process, and LPS-stimulated RAW 264.7 macrophages have been widely used in inflammation studies [1–4]. Several inflammatory mediators such as NO, TNF-α, PGE2, IL-1β and IL-6, as well as the NF-κB signaling pathway, are closely associated with inflammatory diseases [2, 4, 6, 28]. In addition, stimulation of LPS can lead to activation of the NF-κB signaling pathway and result in the production of numerous inflammatory mediators [3–6, 25, 26]. Therefore, suppressing the inflammatory response and reducing the production of inflammatory mediators may be a pivotal strategy for the prevention and treatment of various inflammatory diseases.

NO, produced from eNOS and iNOS, is an important and classic biomarker of inflammation [16, 17]. As one of the most crucial inflammatory mediators, excessive production of NO is an important feature of the inflammatory response of LPS-stimulated RAW 264.7 macrophages [1–4]. In particular, excessive secretion of NO stimulates the activation of NF-κB and other signaling pathways, which leads to the over-secretion of NO, TNF-α, PGE2, IL-1β, IL-6 and other pro-inflammatory cytokines [3–6, 25, 26]. Therefore, inhibiting the overproduction of NO is a vital tool for anti-inflammatory agents. The results of this study showed 19 constituents isolated from *H. plantaginea* flowers significantly suppressed the overproduction of NO in LPS-stimulated RAW 264.7 macrophages. Among them, 5 flavonoids (**1**, **4**–**6** and **15**) and one phenylethanoid glycoside (**17**) exhibited the most significant effect on NO production with IC_50_ values in the range of 12.20–19.91 μM. Comparing the structures of flavonoids and their anti-inflammatory effects, all anti-inflammatory flavonoids contained either zero or two glycosyls except compound **7**, and kaempferol derivatives containing only glucosyls showed stronger anti-inflammatory activities than others belonging to flavonol glycosides, suggesting that glycosylation of certain cites and numbers may contribute to the anti-inflammatory activities of kaempferol. Although many compounds counteract the production of NO in cells, it remains unclear whether eNOS and/or iNOS induce NO.

There is substantial evidence that the massive production of pro-inflammatory cytokines, such as TNF-α, PGE2, IL-1β and IL-6, is closely associated with inflammatory diseases [3–6, 23, 24]. TNF-α participates in the regulation of inflammation and is involved in several inflammatory diseases. Moreover, PGE2 is an important pro-inflammatory mediator that plays a critical role in the course of the inflammatory response. Similarly, IL-1β and IL-6 play a very important role in inflammation. Therefore, suppression of TNF-α, PGE2, IL-1β and IL-6 production seems to be a very effective method to inhibit the abnormal inflammatory response. In this work, compounds **1**, **4**–**6**, **15** and **17** remarkably suppressed the production of TNF-α, PGE2, IL-1β and IL-6 in LPS-activated RAW 264.7 macrophages at a concentration of 20 μM.

Numerous studies have reported that NF-κB is a key transcription factor in the pathogenesis of inflammatory diseases and its activation positively regulates the expression of inflammatory mediators such as NO, TNF-α, PGE2, IL-1β and IL-6 [3–6, 23, 24]. Furthermore, NF-κB consists primarily of p50 and p65 subunits, the latter of which responds to pro-inflammatory cytokine stimulation [5, 28]. Thus, suppressing NF-kB p65 translocation to the nucleus is considered a key target and an effective therapeutic strategy for the treatment of inflammatory diseases. In this study, compounds **1**, **4**–**6**, **15**, and **17** prominently prevented the phosphorylation of p65 translocation, resulting in the blockade of NF-κB subunit p65 nuclear translocation in RAW 264.7 macrophages. These results suggest that the inhibitory effect of these constituents on the NF-κB signaling pathway reduces the levels of inflammatory cytokines, including NO, TNF-α, PGE2, IL-1β and IL-6.

## Conclusions

In conclusion, 5 flavonoids (**1**, **4**–**6** and **15**) and one phenylethanoid glycoside (**17**), especially **4**–**6** derived from the flowers of *H. plantaginea*, exerted significant anti-inflammatory effects by inhibiting the NF-κB signaling pathway and suppressing NO, TNF-α, PGE2, IL-1β and IL-6 in LPS-stimulated RAW 264.7 macrophages. The present study strongly supports the use of *H. plantaginea* flowers as a novel candidate for anti-inflammatory therapy. In addition, these flavonoids and phenylethanoid glycoside may be candidates for the management of inflammatory diseases.

## Supplementary Information

**Additional file 1.**

## Data Availability

The data used to support the findings of this study are available from the corresponding author upon request.

## Abbreviations

ANOVA: One-way analysis of variance
CCK-8: Cell Counting Kit-8
CO_2_: Carbon dioxide
DMEM: Dulbecco’s Modified Eagle’s Medium
ELISA: Enzyme-linked immunosorbent assay
FBS: Fetal bovine serum
LPS: Lipopolysaccharide
IC_50_: Half maximal inhibitory concentration
IL-1β: Interleukin 1β
NO: Nitric oxide
NF-κB: Nuclear factor kappa B
PGE2: Prostaglandin E2
RIPA: Radioimmunoprecipitation assay
SD: Standard deviation
TNF-α: Tumor necrosis factor α
