# Supplementary figures and images for: Anti-inflammatory effects of flavonoids and phenylethanoid glycosides from Hosta plantaginea flowers in LPS-stimulated RAW 264.7 macrophages through inhibition of the NF-κB signaling pathway
Source: BMC Complement Med Ther. 2022 Mar 3;22:55. doi: 10.1186/s12906-022-03540-1 (PMC8895762; doi:10.1186/s12906-022-03540-1)

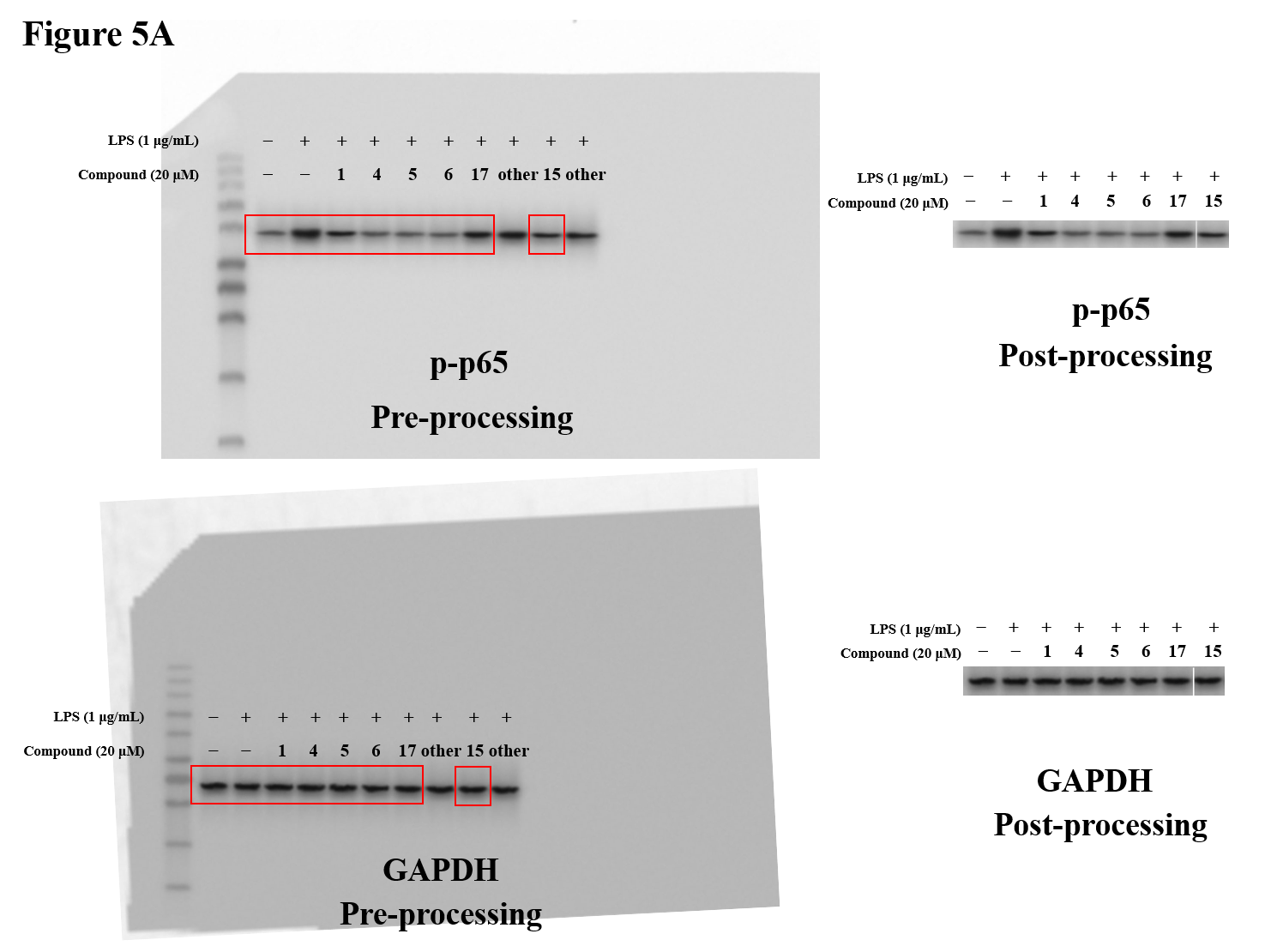


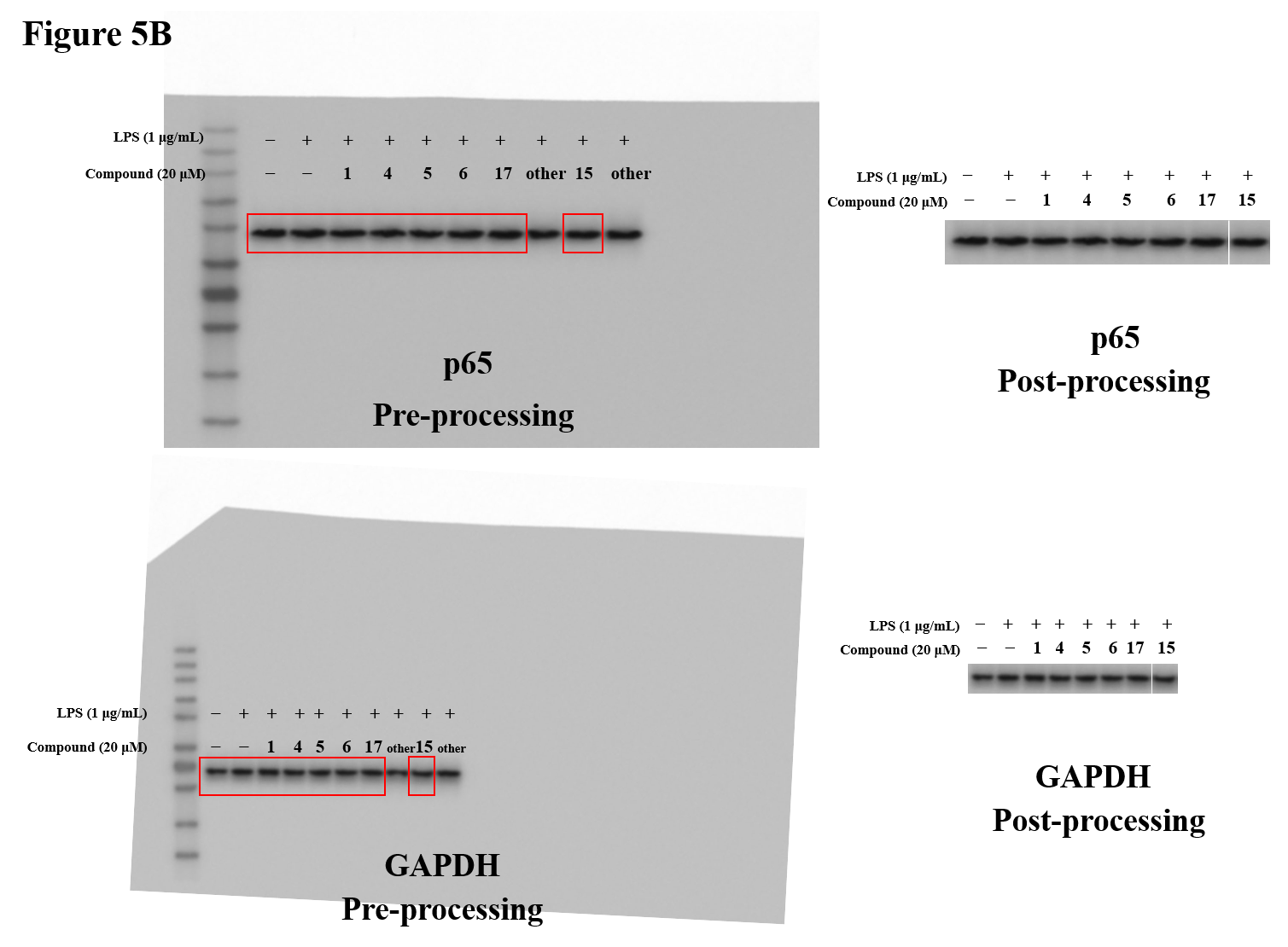

Supplement: Supplementary file 1 — Additional file 1. [file 12906_2022_3540_MOESM1_ESM.docx]
